# Highly selective bile acid hydroxylation by the multifunctional bacterial P450 monooxygenase CYP107D1 (OleP)

**DOI:** 10.1007/s10529-020-02813-4

**Published:** 2020-01-23

**Authors:** Sascha Grobe, Agata Wszołek, Henrike Brundiek, Melinda Fekete, Uwe T. Bornscheuer

**Affiliations:** 1 Department of Biotechnology and Enzyme Catalysis, Institute of Biochemistry, University of Greifswald, 17489 Greifswald, Germany; 2 Enzymicals AG, 17487 Greifswald, Germany

**Keywords:** P450 monooxygenase, Bile acids, Murideoxycholic acid, Lithocholic acid

## Abstract

**Objective:**

Regio- and stereoselective hydroxylation of lithocholic acid (LCA) using CYP107D1 (OleP), a cytochrome P450 monooxygenase from the oleandomycin synthesis pathway of *Streptomyces antibioticus*.

**Results:**

Co-expression of CYP107D1 from *S. antibioticus* and the reductase/ferredoxin system PdR/PdX from *Pseudomonas putida* was performed in *Escherichia coli* whole cells. In vivo hydroxylation of LCA exclusively yielded the 6β-OH product murideoxycholic acid (MDCA). In resting cells, 19.5% of LCA was converted to MDCA within 24 h, resulting in a space time yield of 0.04 mmol L^−1^ h^−1^. NMR spectroscopy confirmed the identity of MDCA as the sole product.

**Conclusions:**

The multifunctional P450 monooxygenase CYP107D1 (OleP) can hydroxylate LCA, forming MDCA as the only product.

**Electronic supplementary material:**

The online version of this article (10.1007/s10529-020-02813-4) contains supplementary material, which is available to authorized users.

## Introduction

Bile acids and their derivatives are steroidal compounds and play an important biological role in digestion by the solubilization of vitamins and fatty acids (Mikov et al. 2006).

Besides their biological function, bile acids have become an interesting compound class for pharmaceutical applications (Hofmann and Hagey 2008). Especially the hydroxylated derivatives of less valuable bile acids, including ursodeoxycholic acid (UDCA), chenodeoxycholic acid (CDCA), and murideoxycholic acid (MDCA) are valuable target molecules.

CDCA (Roda et al. 1982), MDCA (Cohen et al. 1990, 1991) and UDCA (Leuschner et al. 1985) are valuable pharmaceutical agents, because they can be used to solubilize gallstones. Therefore, these bile acid derivatives are applied in treatments of gallstones and liver disease (Chiang 2017).

Up to today the synthesis of valuable bile acids and their derivatives is difficult and the efficiency of the processes is low as reviewed by Tonin and Arends (Tonin and Arends 2018). This is caused by multistep chemical or chemoenzymatic routes with toxic intermediates, protection and deprotection steps to control regio- and stereoselectivity of hydroxylations, and modifications of the steroid nucleus (Eggert et al. 2014).

Cytochrome P450s (CYPs) are capable of hydroxylating otherwise unreactive scaffolds in a regio- and stereoselective fashion and offer the potential to shorten reaction routes towards desirable bile acid derivatives. In the past CYPs have been used to hydroxylate steroidal compounds, like testosterone, in a selective fashion, as reviewed by Fessner and others (Bernhardt 2006; Donova and Egorova 2012; Bernhardt and Urlacher 2014; Fessner 2019).

The P450 monooxygenase CYP107D1, also known as OleP, is part of the oleandomycin synthesis pathway of *Streptomyces antibioticus* (Rodriguez et al. 1995; Shah et al. 2000). In this pathway it selectively introduces an epoxide functionality in a macrolide (Montemiglio et al. 2016; Parisi et al. 2018). More importantly, CYP107D1 has been reported to unspecifically hydroxylate testosterone (Agematu 2006).

We decided to investigate the ability of CYP107D1 to hydroxylate the bile acids lithocholic acid (LCA) and deoxycholic acid (DCA) to more valuable derivatives. We constructed a whole-cell biotransformation process based on *E. coli* co-expressing CYP107D1 and the PdR/PdX reductase system from *Pseudomonas putida*. We found that CYP107D1 stereo- and regioselectively hydroxylates LCA and DCA at the 6β-position. This converted the less valuable secondary bile acid LCA to the more valuable MDCA, without the formation of any side products. The P450 monooxygenase OleP is therefore a promising enzyme for the production of bile acid derivatives.

## Material and methods

### Strains, expression vectors, enzymes, and chemicals

*Escherichia coli* C43 (DE3), purchased from Lucigen, was used for expression and whole-cell biotransformations. The plasmid containing the genes for the redox partners *PdR* and *PdX* were provided by Prof. Anett Schallmey (University of Braunschweig). CYP107D1 was purchased from GenScript as codon-optimized synthetic gene based on the GenBank entry Q59819 and was subcloned into pET-28a vector. All chemicals and solvents were purchased from Sigma Aldrich and used without further purification. LCA was purchased from ACROS and DCA from Sigma Aldrich both with a purity of 98% or higher.

### Whole-cell biocatalysis

For whole-cell biocatalysis under optimized conditions, an overnight culture of LB media (25 µg mL^−1^ kanamycin, 25 µg mL^−1^ chloramphenicol) was inoculated from a glycerol stock of *E. coli* C43 (DE3) co-transformed with the plasmids pET-28a-*oleP* and pACYC-*pdR/pdX* and grown for 16 h at 37 °C. TB medium (25 µg mL^−1^ kanamycin, 25 µg mL^−1^ chloramphenicol) was inoculated with 0.1% (ν/ν) of the overnight culture and grown at 37 °C until an OD_600_ of 0.7 was reached. Subsequently, the culture was cooled to 28 °C and supplemented with 0.64 mM δ-aminolaevulinic acid and 0.3 mM FeSO_4_. Protein expression was then induced using 0.4 mM IPTG (isopropyl-β-d-thiogalactopyranoside) and the culture was incubated for another 20 h at 28 °C. Cells were harvested by centrifugation (3000×*g*, 45 min, 4 °C) and resuspended in resting cell medium (200 mM KH_2_PO_4_/K_2_HPO_4_, 20 mM NaCl, 1% (*w/v*) glucose, 0.4% (*w/v*) glycerol, pH 7.4) and adjusted to a cell dry weight of 6.6 g L^−1^ (Glazyrina et al. 2010). Substrate was added to a final concentration of 2 mg mL^−1^ from a 100 mg mL^−1^ stock solution in DMSO. Reactions were incubated at 28 °C with shaking at 160 rpm. Samples (1 mL) were taken over a period of 24 h and centrifuged (13,300×*g*, 5 min) to remove cells. The supernatants were used for quantification of the bile acids.

### Analytics

Samples (1 mL) were extracted twice with 1 mL ethyl acetate. The combined organic phases were dried over anhydrous Na_2_SO_4_. After complete evaporation of ethyl acetate, the residue was dissolved in 200 µL ethanol. Samples (20 µL) were then analyzed using a Hitachi LaChrom Elite® HPLC System (Hitachi High-Technologies, Krefeld, Germany) with a Luna® Omega 5 µm/100 Å PS C18 column (Phenomenex, Aschaffenburg, Germany). The mobile phase was acetonitrile:water (50:50)% (v/v) containing 0.1% trifluoroacetic acid. An isocratic method (1 mL min^−1^) was employed at room temperature. The bile acids were detected using a LaChrom Elite L-2490 RI Detector (Hitachi High-Technologies, Krefeld, Germany).

### Preparative whole-cell biocatalysis

For the preparative whole-cell biocatalysis, cells corresponding to a dry cell weight of 50 g L^−1^ were resuspended in resting cell medium. Bile acids were added to a final concentration of 1 mg mL^−1^ (stock solutions were 10 mg mL^−1^ bile acid dispersed in water). After 24 h, the biotransformation mixture was worked up and the product was isolated via column chromatography (see details in Supporting information). The obtained product was an off-white powder.

### NMR spectroscopy

NMR spectra were recorded with a Bruker Avance III HD 600 spectrometer equipped with an inverse ^1^H/^13^C/^15^N/^31^P quadruple resonance cryoprobe head and z-field gradient (Bruker BioSpin GmbH, Rheinstetten, Germany). Products were dissolved in deuterated methanol and identification was based on 2D-NMR techniques.

## Results and discussion

### Biotransformation of LCA using OleP and PdR/PdX

**Fig. 1:**
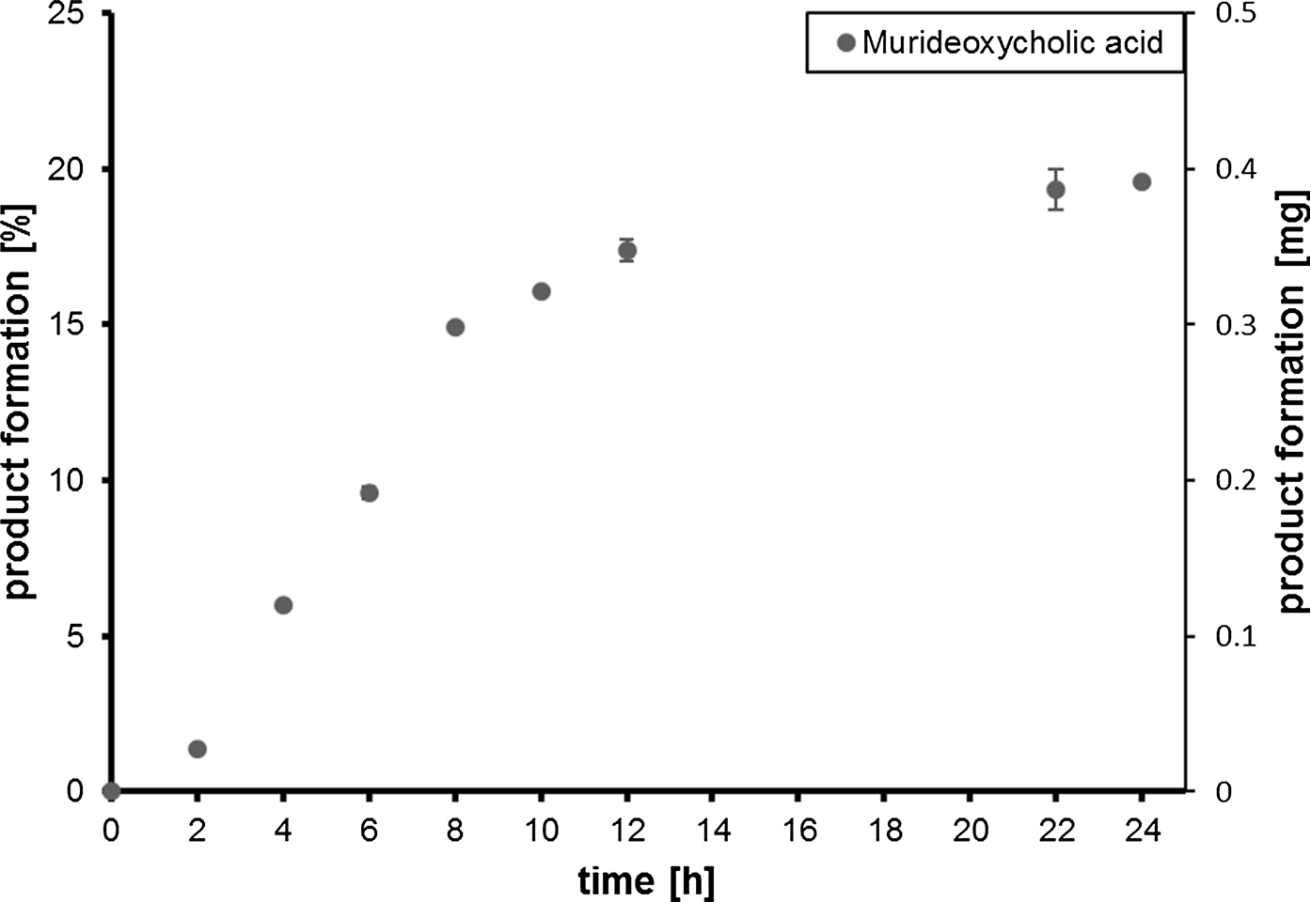
Whole-cell biotransformation of 5.31 mM LCA using *E. coli* C43 (DE3) co-expressing CYP107D1 (OleP) and PdR/PdX. The percentage and amount (in mg) of LCA converted to MDCA at each time point is plotted as mean with standard deviation (n = 3)

Whole cells of *E. coli* C43 (DE3) co-expressing OleP (CYP107D1) and PdR/PdX converted LCA (2 mg mL^−1^, 5.31 mM) to a single product according to HPLC analysis. To identify the product, the reaction was repeated on preparative scale, monitored over 24 h, and products were analyzed by NMR spectroscopy (Supplementary Figs. 1, 2, 3 and 7). This revealed MDCA as the sole product (Fig. 1). Most of the MDCA product formation is seen within 8–12 h.

Within 24 h, 0.39 mg mL^−1^ (0.99 mM) MDCA were produced, resulting in a yield of 19.5% MDCA. Consequently, the space time yield of MDCA formation is 0.042 mM h^−1^.

Previously CYP107D1 was only described for its involvement in the synthesis of oleandomycin (Montemiglio et al. 2016; Parisi et al. 2018) and forming the 6β-, 17β-, 12β-, and 15β-hydroxylated derivatives of testosterone (Agematu et al. 2006). To the best of our knowledge, the selective 6β-hydroxylation of LCA by CYP107D1 (OleP) had not been described. We were able to show that MDCA was formed as sole product by feeding *E. coli* whole cells with LCA.

The formation of MDCA as one of several products from LCA is described for mammalian CYPs including CYP3A10 in transfected COS cells and several studies using microsomes (Teixeira and Gill 1991; Deo and Bandiera 2008, 2009). Compared to CYP107D1 most of these reactions result in several hydroxylated species derived from LCA (Zimniak et al. 1989; Dionne et al. 1994). With a sequence similarity of only 20%, CYP3A10 and CYP107D1 are not closely related, which explains the high selectivity of CYP107D1. Using CYP107D1 with *E. coli* as expression host is thus a promising future process for the selective hydroxylation compared to biotransformations using CYP3A10.

### Expanding the scope towards deoxycholic acid (DCA)

To further verify if the selective hydroxylation by CYP107D1 occurs also with other bile acids, DCA was used as further substrate (for a list of all investigated substrates see Supporting Information, Table 1). DCA is also part of the pool of less valuable secondary bile acids. The structural difference between LCA and DCA is the additional 12α-hydroxyl group which changes the hydrophilic-lipophobic balance of the bile acid leading to an overall higher hydrophilicity of the compound (Monte et al. 2009). When DCA was used in a whole cell biotransformation at 200 mL scale with 200 mg DCA, the single obtained product was isolated in 35% yield after column chromatography. Its structure was identified by NMR spectroscopy to be 3α-, 6β-, 12α-trihydroxy-5β-cholan-24-oic acid, which is the 6β-hydroxylated product of deoxycholic acid (Supplementary Figs. 4, 5, 6). The synthesis of 6β-hydroxylated DCA starting from DCA was previously described in 11 steps with an overall yield below 20% (Iida et al. 1991). This further indicates CYP107D1 as beneficial candidate for future processes compared to multistep chemical pathways.

## Conclusions

We could show that CYP107D1 is able to selectively hydroxylate the secondary bile acids LCA and DCA to the corresponding 6β-OH products MDCA and 3α, 6β, 12α-trihydroxy-5β-cholan-24-oic acid. The product formation was followed over 24 h and the products were confirmed by NMR spectroscopy. We thus achieved the formation of the valuable compound MDCA which has gallstone solubilization properties (Cohen et al. 1990, 1991). The specificity of the hydroxylation using CYP107D1 provides a valuable entry point for the late stage modification of secondary bile acids and holds promise for applications in biotechnology and medicinal chemistry.

## Electronic supplementary material

Below is the link to the electronic supplementary material.

Supplementary file1 (DOCX 439 kb)
