# Supplementary material for: Highly selective bile acid hydroxylation by the multifunctional bacterial P450 monooxygenase CYP107D1 (OleP)
Source: Biotechnol Lett. 2020 Jan 23;42(5):819–24. doi: 10.1007/s10529-020-02813-4 (PMC7101289; doi:10.1007/s10529-020-02813-4)
Supplement: Supplementary file 1 — Supplementary file1 (DOCX 439 kb) [file 10529_2020_2813_MOESM1_ESM.docx]

Supporting Information

**Work-up of preparative scale biotransformations and isolation of products**

The biotransformation mixture was acidified with concentrated H_2_SO_4_ to pH 2. Cells were separated from the broth via centrifugation and washed with acetone, then filtered. The aqueous fermentation broth was extracted 4x with ethyl acetate. The combined organic fractions were dried over MgSO_4_ and evaporated. The product was isolated from the crude mixture via column chromatography on silica gel. Eluent: dichloromethane: acetone: acetic acid 60:30:1. The 6β-hydroxylated bile acids appeared as off-white powder.

**Product quantification/substrate conversion by HPLC-RI**

The quantification of the observed products was done by HPLC-RI using the by NMR-spectroscopy confirmed off white powder resulting from the preparative scale biotransformations of MDCA as well as commercially available LCA. Different concentration ranging from 4 mg·mL^-1^ to 0.1 mg·mL^-1^ of MDCA and LCA were prepared and measured by HPLC-RI. The resulting equation was used to calculate the amount of produced MDCA over time. The internal standard was used to evaluate the quality of the extraction process. The results of the standard series are displayed in the Supplementary Figures 8 and 9.

**Supplementary Figure 1**: Numbering of MDCA according to the IUPAC nomenclature.

3α,6β-dihydroxy-5β-cholan-24-oic acid. [M^-^]: 391.5; ^1^H-NMR *δ* (ppm, MeOD): 0.72 (s, 18-Me), 1.10 (s, 19-Me), 3.51 (tt, J=4.7, 11Hz, 3-H), 3.69 (H-6); ^13^C-NMR *δ* (ppm, MeOD): 12.6 (C-18), 26.16 (C-19), 72.1 (C-3), 74.04 (C-6)

**
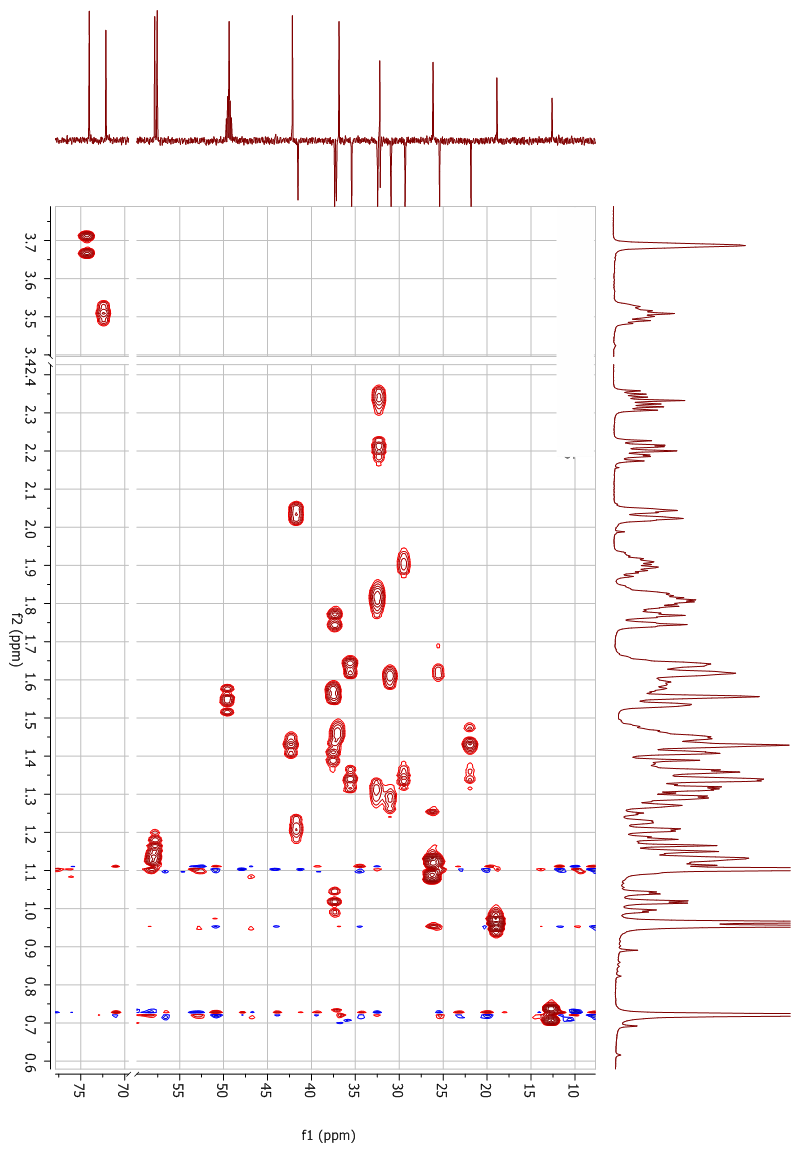
**

**Supplementary Figure 2**: 2D-NMR of the purified MDCA (3α,6β-dihydroxy-5β-cholan-24-oic acid).

**
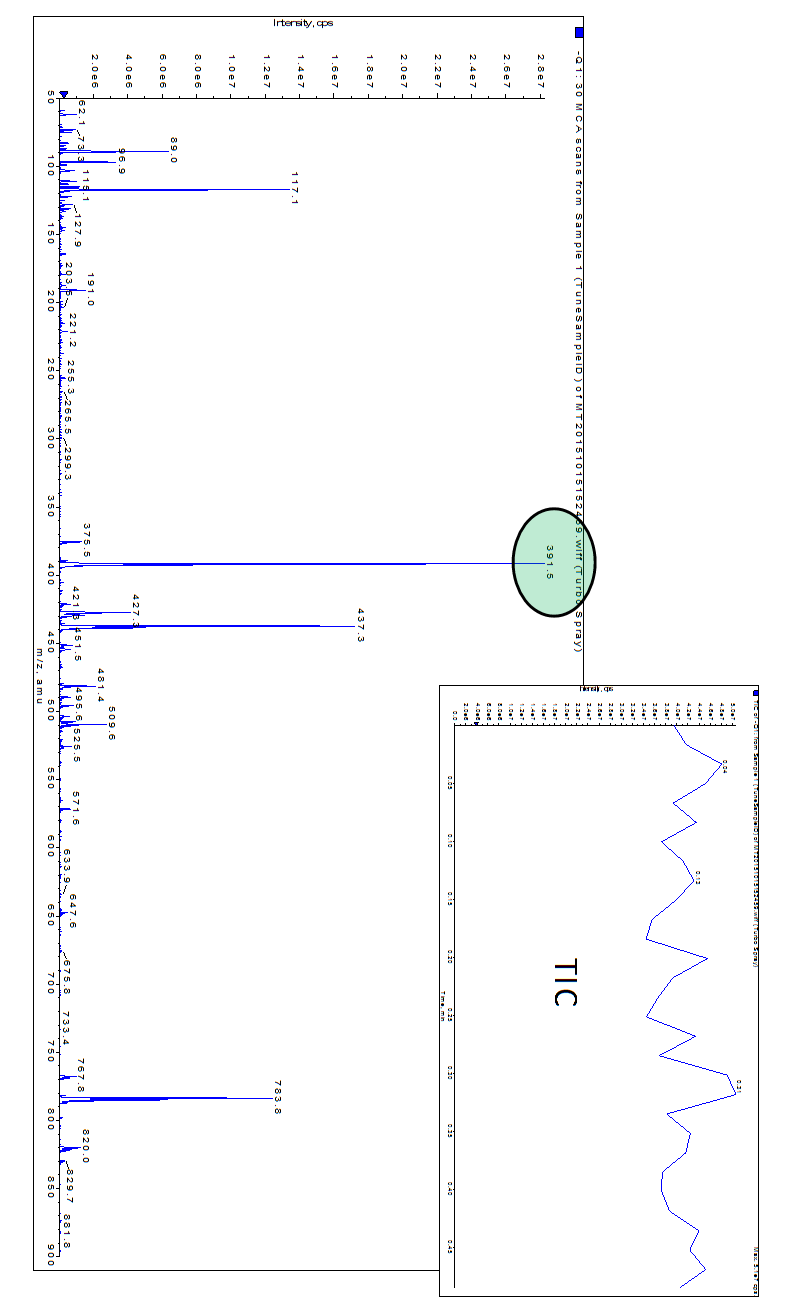
**

**Supplementary Figure 3:** MS verification of MDCA.

**Supplementary Figure 4:** Numbering of 3α,6β,12α-trihydroxy-5β-cholan-24-oic acid according to the IUPAC nomenclature.

3α,6β,12α-trihydroxy-5b-cholan-24-oic acid. [M^-^]: 407.4; 1H-NMR δ (ppm, MeOD): 0.74 (s, 18-Me), 1.09 (s, 19-Me), 3.50 (tt, J=4.7, 11Hz, 3-H), 3.69 (q, J=2.60Hz, H-6), 3.98 (t, J=2.70Hz, H-12); 13C-NMR δ (ppm, MeOD): 13.80 (C-18), 26.40 (C-19), 72.83 (C-3), 74.55 (C-6), 74.60 (C-12).

**
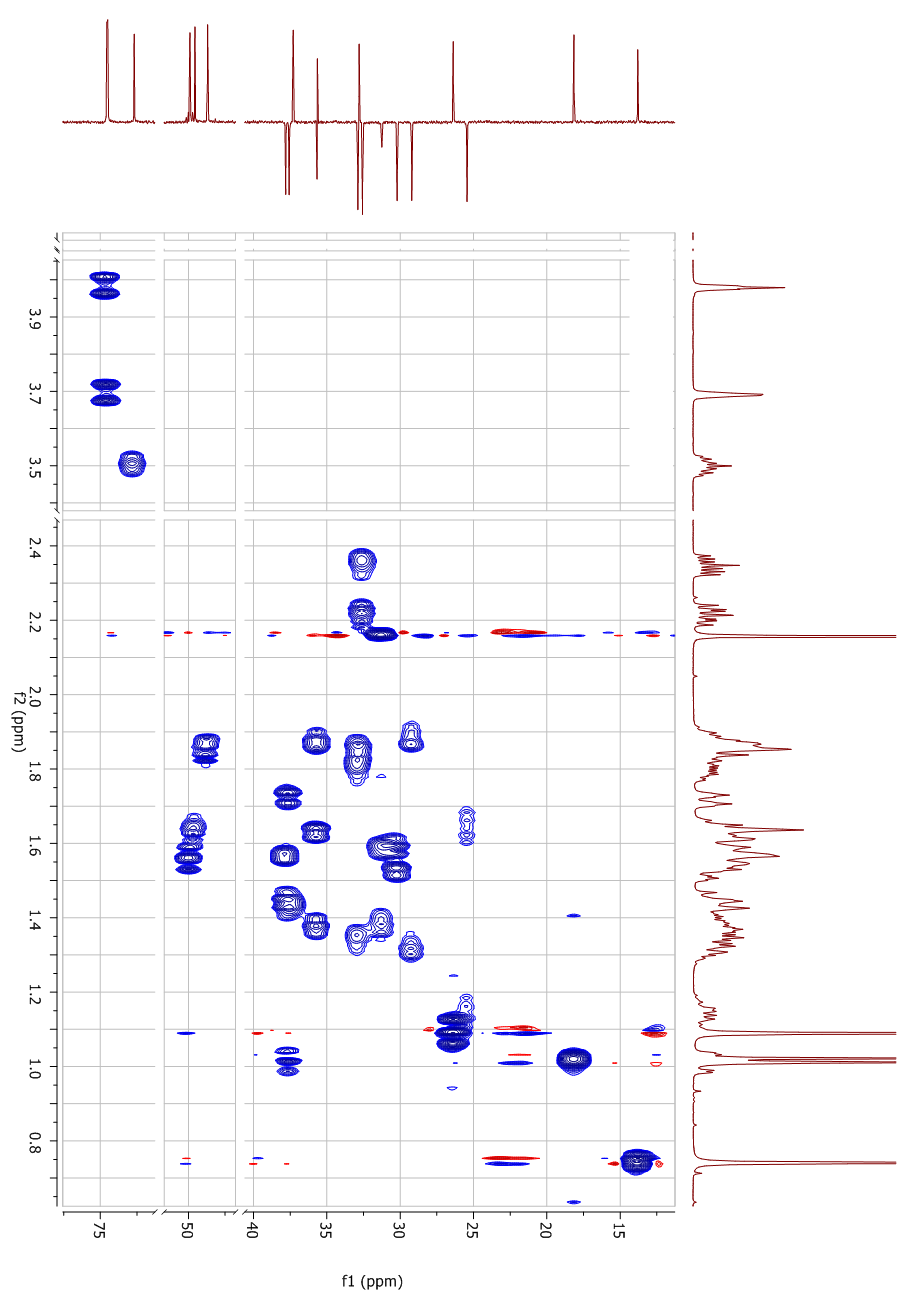
**

**Supplementary Figure 5:** 2D NMR of the purified 3α,6β,12α-trihydroxy-5β-cholan-24-oic acid.

**Supplementary Figure 6**: MS verification of 3α,6β,12α-trihydroxy-5β-cholan-24-oic acid.


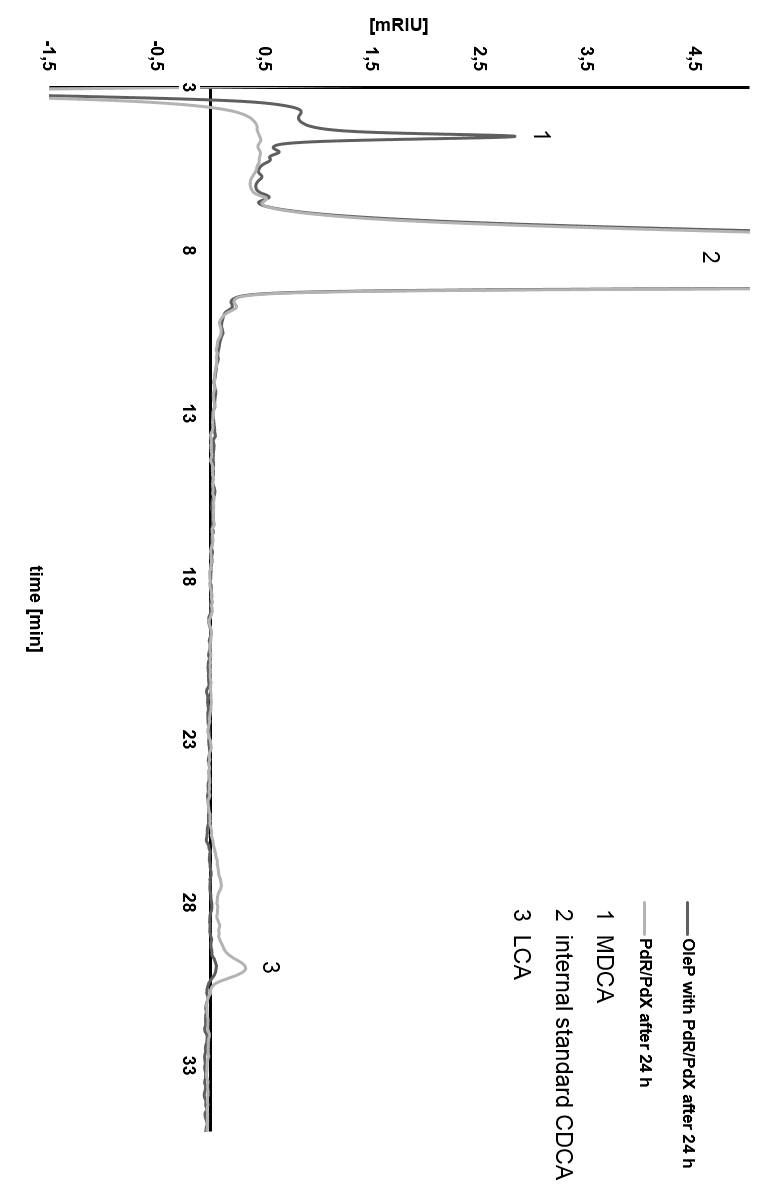


**Supplementary Figure 7**: HPLC-RI chromatograms of *E. coli* C43 (DE3) pET-28a *oleP*, pACYC *pdR/pdX* (black) and *E. coli* C43 (DE3) pACYC *pdR/pdX* (grey) are overlayed. Peaks marked with numbers 1 correspond to MDCA, 2 corresponds to the internal standard used CDCA and 3 corresponds to LCA.

**Supplementary Figure 8:** Product quantification of MDCA based on HPLC-RI measurements.

**Supplementary Figure 9:** Substrate quantification of LCA based on HPLC-RI measurements.

**Supplementary Table 1**: Tested bile acid for conversion using CYP107D1.

CYP107D1 was tested with the following bile acids as substrate for activity of which only LCA and DCA showed conversion.

| **No** | **Substrate** | **Systematic name** | **Con-version** |
| --- | --- | --- | --- |
| 1 | Cholanic acid | 5β-cholan-24-oic acid | **-** |
| 2 | Lithocholic acid (LCA) | 3α-hydroxy-5β-cholan-24-oic acid | **+** |
| 3 | Allolithocholic acid | 3α-hydroxy-5α-cholan-24-oic acid | **-** |
| 4 | Chenodeoxycholic acid (CDCA) | 3α,7α-dihydroxy-5β-cholan-24-oic acid | **-** |
| 5 | Deoxycholic acid (DCA) | 3α,12α-dihydroxy-5β-cholan-24-oic acid | **+** |
| 6 | Hyodeoxycholic acid (HDCA) | 3α,6α-dihydroxy-5β-cholan-24-oic acid | **-** |
| 7 | Ursodeoxycholic acid (UDCA) | 3α,7β-dihydroxy-5β-cholan-24-oic acid | **-** |
